# Genotyping-by-sequencing highlights patterns of genetic structure and domestication in artichoke and cardoon

**DOI:** 10.1371/journal.pone.0205988

**Published:** 2018-10-23

**Authors:** Stefano Pavan, Pasquale L. Curci, Diana L. Zuluaga, Emanuela Blanco, Gabriella Sonnante

**Affiliations:** 1 Department of Soil, Plant and Food Science, University of Bari ˝Aldo Moro˝, Bari, Italy; 2 Institute of Biomedical Technologies, National Research Council (CNR), Bari, Italy; 3 Institute of Biosciences and Bioresources, CNR, Bari, Italy; Washington University, UNITED STATES

## Abstract

Exploiting the biodiversity of crops and their wild relatives is fundamental for maintaining and increasing food security. The species *Cynara cardunculus* includes three taxa: the globe artichoke, one of the most important Mediterranean vegetables, the leafy cardoon, and the wild cardoon. In this study, genotyping by sequencing (GBS) was successfully applied to reveal thousands of polymorphisms in a *C*. *cardunculus* germplasm collection, including 65 globe artichoke, 9 leafy cardoon, and 21 wild cardoon samples. The collection showed a strong population structure at K = 2, separating the globe artichoke from the leafy and wild cardoon. At higher K values, further substructures were observed, in which the wild cardoon was separated from the leafy cardoon, and the latter included the Spanish wild cardoons, while the wild sample from Portugal was admixed. Moreover, subpopulations within the globe artichoke set were highlighted. Structure analysis restricted to the globe artichoke dataset pointed out genetic differentiation between the ˝Catanesi˝ typology and all the other samples (K = 2). At higher values of K, the separation of the ˝Catanesi˝ group still held true, and green headed landraces from Apulia region, Italy (˝Green Apulian˝) formed a distinct subpopulation. ˝Romaneschi˝ artichoke types fell in a variable group with admixed samples, indicating that they should not be considered as a genetically uniform typology. The results of principal component analysis and Neighbor-Joining hierarchical clustering were consistent with structure results, and in addition provided a measure of genetic relationships among individual genotypes. Both analyses attributed the wild material from Spain and Portugal to the cultivated cardoon group, supporting the idea that this might be indeed a feral form of the leafy cardoon. Different reproductive habit and possibly selective pressure led to a slower LD decay in artichoke compared to cardoon. Genotyping by sequencing has proven a reliable methodology to obtain valuable SNPs and assess population genetics in *C*. *cardunculus*.

## Introduction

The analysis of genetic variation of crop species and their wild relatives is a crucial aspect for biodiversity conservation and its exploitation to maintain and increase food security [1]. Nowadays, the exploration of plant biodiversity is boosted by advanced sequencing technologies, which provide the opportunity to simultaneously discover and test a high number of molecular markers at a relatively low cost. In particular, genotyping by sequencing (GBS), based on high-throughput sequencing of a reduced complexity genome library generated by restriction enzymes, proved to be cost-effective in the development and genotyping of thousands of single nucleotide polymorphism (SNP) markers. So far, GBS has been extensively used in species with or without an available reference genome [2, 3] for linkage map construction [4, 5], genomic selection [6], and the exploration of plant genetic diversity on a genome-wide scale [3, 7–9].

The globe artichoke [*Cynara cardunculus* var. *scolymus* (L.) Fiori] is conspecific to and interfertile with the cultivated leafy cardoon (*C*. *cardunculus* var. *altilis* DC), and the wild progenitor of the two crops (*C*. *cardunculus* var. *sylvestris* Lam.). Therefore, the latter two entities belong to the artichoke primary gene pool [10, 11, 12, 13]. The globe artichoke, a diploid plant (2n  =  2x  =  34) with an estimated genome size of 1.07 Gb [14], is a typical Mediterranean vegetable crop, mainly consumed for its immature flower heads and traditionally cultivated in southern Europe and Northern Africa, with a more recent diffusion in California, South America, and China. Both heads and leaves are rich in antioxidant phenolic compounds such as flavonoids, caffeic acid, chlorogenic acid and cynarin, and for this reason, artichoke plants are used in herbal and medicinal preparations [15–17]. Moreover, artichoke organs, including roots, contain a high amount of inulin molecules [18] with a chain length of up to 200 [19], having a marked and well tolerated prebiotic effect in humans and useful for industrial applications, e.g. for inhibiting the growth of ice crystals in frozen foodstuffs [20].

In general, the globe artichoke is clonally reproduced to ensure commercial uniformity [21], although seed-propagated varieties have been released in recent years [22]. The morphological diversity in head shape and colour, coupled with flowering time, led to the distinction of four main groups, namely ˝Catanesi˝, ˝Romaneschi˝, ˝Spinosi˝, and ˝Violetti˝ [23]. However, not all the traditional varieties fall in these groups, and molecular markers have often failed to attribute a specific membership for some of them [21, 24]. Italy possesses the richest germplasm diversity of globe artichoke, resulting in the local cultivation of many landraces, well adapted to local climatic conditions [12, 23, 25]. Leafy cardoon is grown on a small scale in northern Italy, southern France and in Spain for its large leaf stalks [11].

All the genus *Cynara*, and the species *C*. *cardunculus* in particular, originated in the Mediterranean area. Probably, the globe artichoke was domesticated in Sicily, while the cultivated cardoon (CC) followed a distinct domestication pathway possibly in the Iberian Peninsula and the South of France [10, 11]. In fact, the leafy cardoon was selected for the gigantism of leaf stalks, which are used as vegetables to prepare traditional dishes [11, 21]. The wild cardoon, the progenitor of both *C*. *cardunculus* crops, is distributed across the Mediterranean Region, from Cyprus and the Black Sea to Atlantic Spain, Portugal and the Canary Islands [26]. Its small flower heads are traditionally gathered from the wild and used as a food in southern Italy [27]. Although two wild forms with distinct morphology and distribution (eastern and western-Mediterranean) were distinguished [11, 24, 26], SSR analysis and population structure suggested that the western wild cardoon could be a feral form and not a real wild type [21].

Recently, a draft of the globe artichoke genome was obtained by Illumina sequencing, producing 13,588 scaffolds covering 725 Mb of the genome [28], and resequencing was performed on four artichoke genotypes and one cultivated cardoon [29]. The availability of a reference genome allows the mapping of short reads obtained from GBS analysis to the genome, allowing the application of accurate SNP calling pipelines [30].

In the present study, we used for the first time the GBS approach to explore genome-wide SNP variation in a *C*. *cardunculus* collection including globe artichoke traditional varieties, cultivated leafy cardoon and wild cardoon. The aim of this work was to detect population genetic structure, patterns of genetic diversity, and relationships in artichoke and its conspecific allies.

## Material and methods

### Plant material

A group of 95 *C*. *cardunculus* samples was prepared for this study, including 65 artichokes, 9 cultivated cardoons, and 21 wild cardoons (S1 Table). Samples (artichoke and wild material) were obtained from the *Cynara* collection held at the Institute of Biosciences and BioResources (CNR, Bari, Italy) or from exchange with other institutions (GEVES, Cavaillon France; Botanic Institute, CSIC, Barcelona, Spain; ITGA, Navarra, Spain; University of Tunis, Tunisia). For the artichoke samples, the four main morpho-agronomic groups were considered: the small headed early types (˝Catanesi˝), the spiny early types (˝Spinosi˝), the violet late types (˝Violetti˝), and the large headed late types (˝Romaneschi˝) [23]. Several other accessions, belonging to none of the above typologies, were analysed [12, 31]. The cultivated cardoon germplasm included material originating from the main regions where this vegetable is grown, i.e. Spain, France, and Italy. The wild material was selected in order to encompass the geographical distribution of the taxon.

### GBS assay and SNP filtering

Genomic DNA was isolated from young leaves as previously described [32]. A *Pst*I library was prepared at the Institute for Genomic Diversity at Cornell University (http://www.biotech.cornell.edu/), according to the method of Elshire et al. [2], except for the use of *Pst*I enzyme and corresponding adapters. The library was sequenced at the Cornell University (Ithaca, U.S.A.), with the Illumina technology using a HiSeq2500 in high output mode (single-end 100 bp reads), as a single lane including an empty, negative control well. The sequencing reads were searched for 100% matching barcode with the expected bases remnant of the enzyme cut site. The reads containing the barcode were sorted, de-multiplexed and trimmed to first 64 bases starting from enzyme cut site. After this step, the reads containing "N" within the first 64 bases were rejected. The TASSEL-GBS pipeline [33], implementing the globe artichoke genome assembly (NCBI SRA project number PRJNA238069) and the Burrows-Wheeler Aligner (version 0.7.8-r455) algorithm [34], was used to call SNPs and generate a vcf variant file. Minor allele frequency (MAF) higher than 5%, call rate higher than 50%, and Hardy-Weinberg equilibrium p-value >10^−6^ [35] was applied for further filtering biallelic SNPs, using TASSEL v5.2.20 [36] and SNP & Variation Suite (SVS) software v8.4.0 (Golden Helix Inc., Bozeman, MT, U.S.A.). Genotypes with more than 30% missing data were removed from further analysis. The filtering procedure was applied to the whole germplasm collection and to separate subsets including either globe artichokes or cardoons. The VCFtools package [37] and TASSEL5.2.20 [36] were used to derive the distribution of SNP substitution types and the proportion of heterozygous loci.

### Population structure analysis, genetic diversity and relationships

A structure analysis was carried out using the Bayesan clustering approach and the admixture model implemented in the software STRUCTURE (ver. 2.3.4) [38]. For a number of hypothetical subpopulations (the K parameter) varying from 2 to 10, ten independent runs were performed applying 50,000 burn-in period and 100,000 Markov Chain Monte Carlo (MCMC) repetitions. Prior to analysis, the SNP dataset was pruned based on pairwise linkage disequilibrium (LD) between adjacent markers, using SVS v8.4.0 and setting the threshold for r^2^ equal to 0.5.

The most probable K value was determined by *ad hoc* ΔK statistics [39], using the software Structure Harvester [40]. Individual samples were assigned to each subpopulation when the value of the corresponding membership coefficient (q) was higher than 0.75, otherwise they were considered admixed.

Expected heterozygosity between individuals in the same cluster and allele frequency divergence, ˝net nucleotide distance˝ between clusters, were obtained from STRUCTURE analysis.

SNPs selected as described above were also used to evaluate genetic relationships among genotypes within the whole dataset and the subsets of artichokes and cardoons. In particular, principal component analyses (PCA) were carried out using SVS v8.4.0., whereas Neighbor-Joining (NJ) [41] trees, based on the Tamura-Nei genetic distance model [42], were built after performing five hundred bootstrap replicates using MEGA7 package (https://www.megasoftware.net/).

### LD decay

The extent of LD decay was assessed for the whole collection, for the globe artichoke dataset, and for the cardoon group, separately. Pairwise r^2^ values were estimated using the expectation-maximization (EM) algorithm implemented in SVS v8.4.0 and then plotted against the distance (kb) between adjacent SNP loci.

## Results

### GBS experiment and SNP calling

Sequencing of a 95-plex GBS library of *C*. *cardunculus* yielded about 259 million reads. Of these, about 230 million were considered of good quality, exhibiting both a full barcode and the expected remnant of the restriction cut site [2]. Tags (unique sequences of 64 bp following the barcode) occurring at least three times were over 2.3 million, of which 27.6% were successfully mapped (25.9% uniquely and 1.7% multiply), while 72.4% remained unaligned. A master variant calling file with 22,883 SNPs was produced with the TASSEL-GBS pipeline. After filtering SNPs and samples as described in the Methods section, a final dataset of 92 samples and 3,762 SNPs was obtained (S2 Table). Filtering based on Hardy-Weinberg equilibrium (p>10^−6^) successfully eliminated highly heterozygous loci, which are expected in case of sequencing errors or paralogous loci (S1 Fig). The observed transition/transversion rate was 1.24. The most abundant substitution type was A/G with 28.7%, while the lowest represented class was C/G with 4.9% (S2 Fig).

The filtering procedure was repeated on sample subsets containing just the globe artichokes or the wild cardoons, leading to the identification of 4,711 (62 genotypes) and 6,668 (30 genotypes) SNPs markers, respectively (S2 Table).

Mean observed heterozygosity was 31.2% for the globe artichoke collection, and 15.2% for the cardoon dataset.

### Genetic structure

**Fig 1:**
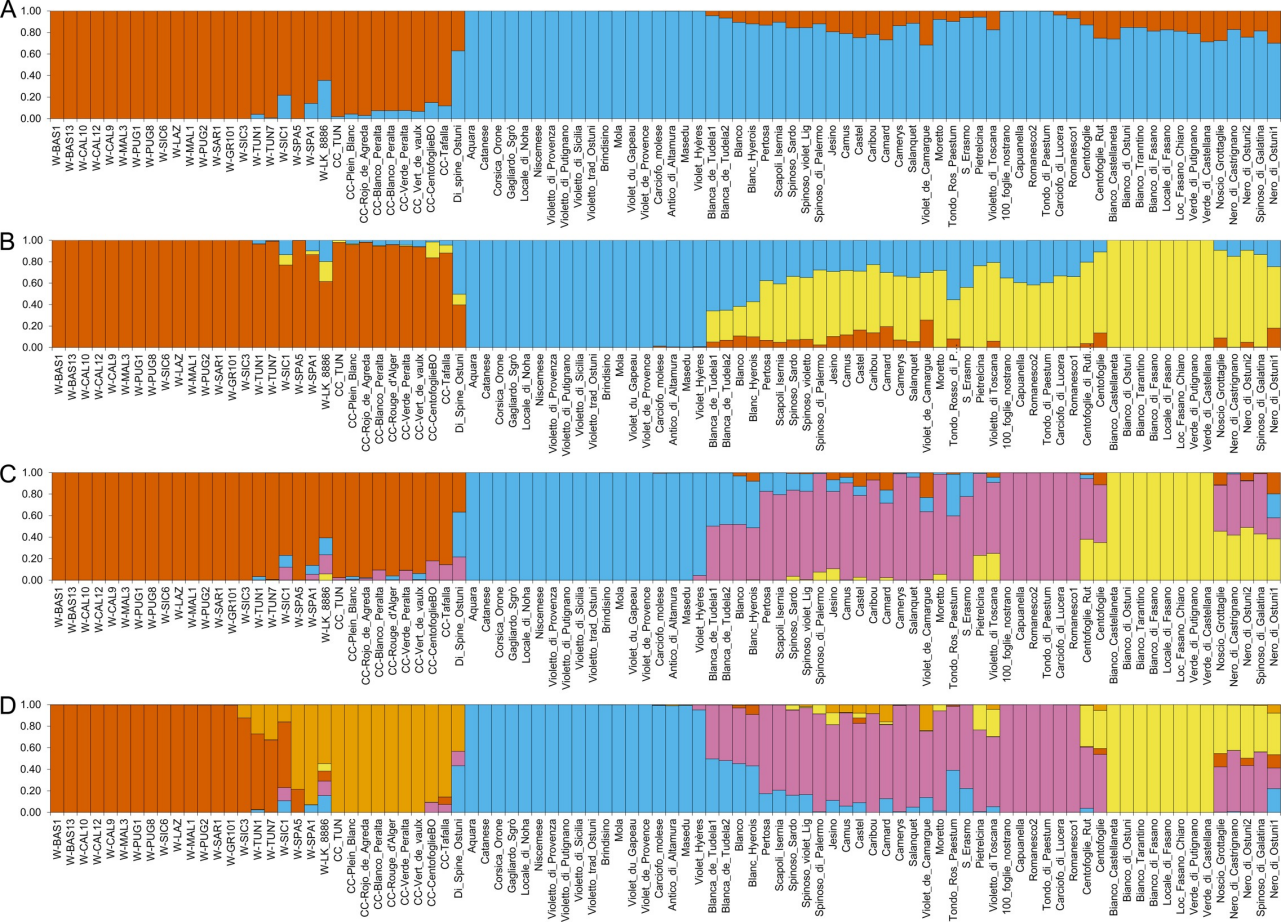
Structure analysis of *Cynara cardunculus* genotypes. Clusters inferred from the population structure analysis at (A) K = 2, (B) K = 3, (C) K = 4, (D) K = 5. Numbers on the y-axis show the subgroup membership. Genotype names are reported on the x axis. The colours of the bars indicate the groups identified through the STRUCTURE program.

The genetic structure of the *C*. *cardunculus* collection was investigated by means of the admixture model implemented in the software STRUCTURE. The Evanno delta K method identified the best model as the one containing two subpopulations (K = 2), followed by those with K = 3 and K = 4 (S3 Fig). Based on the K = 2 model, genotypes cluster according to their taxonomic distribution, with groups corresponding to globe artichoke samples on one side, and wild and cultivated cardoons on the other side (Fig 1A). The individual membership coefficient to each population (q) is very high, most cardoons and artichokes showing a ˝q˝ above 0.9 and 0.85, respectively. However, the Portuguese cardoon sample (W_LK_8886) and seven artichoke samples display a ˝q˝ value below 0.75, indicating an admixed ancestry. The artichoke sample with the lowest ˝q˝ value (0.63) corresponds to the landrace ˝Di spine Ostuni˝. Although this genotype is considered as a globe artichoke by the donor farmer, it has a flower head morphology resembling cultivated cardoons, and might therefore have derived from a hybridization event between the two taxa.

Since the Evanno method can underestimate K when there is hierarchical population structure [43], we also considered STRUCTURE models with K>2. At K = 3 (Fig 1B), wild and cultivated cardoons are still joined in the same group, and the Portuguese cardoon appears admixed (q = 0.62). The globe artichokes are split into two main groups, one including the ˝Catanesi˝ types (q = 0.99–1.00), and the other containing green headed artichokes from Apulia region in Southern Italy (˝Green Apulian˝ artichokes, with q = 1.00) and other genotypes (q ranging from 0.75 to 0.86). Moreover, many admixed genotypes can be observed, including ˝Romaneschi˝, ˝Spinosi˝, most of the French artichokes, and Blanca de Tudela. In the K = 4 solution (Fig 1C), besides the clusters observed at K = 3, a distinct artichoke group arises, composed of ˝Romaneschi˝, ˝Spinosi˝ and ˝Violetti˝ varieties (dark pink in Fig 1C). The K = 5 outcome separates the wild (dark orange in Fig 1D) from the cultivated (light orange in Fig 1D) cardoons, with the wild cardoons from Spain included in the group of cultivated cardoons. Some wild genotypes are admixed, namely W_LK_8886 (mostly between CC and artichoke groups), one cardoon from Sicily (with a higher ˝q˝ value for the wild group and lower contributions of CC and artichoke groups), and two cardoons from Tunisia (with a higher ˝q˝ value for the wild group and some CC contribution). The artichoke samples are organized in groups resembling those for K = 4, however several genotypes are admixed. Some of them share a higher ˝q˝ value for the Romaneschi/Spinosi/Violetti group (e.g. Camard, Castel, Jesino, Violetto di Toscana), whereas others display higher degree of admixture, such as Blanca de Tudela, sharing approximately equal ancestry between the ˝Catanesi˝ and the ˝Romaneschi˝ groups. At K = 6 (S4 Fig), some ˝Romaneschi˝ genotypes (100_foglie, Capuanella, Carciofo di Lucera, Romanesco, Tondo di Paestum) form a cluster distinct from other ˝Romaneschi˝ types (e.g. Camus, Castel, Jesino, etc.), which are grouped with the ˝Spinosi˝ types and the landrace Violetto di Toscana. Increasing the K value to 7 (S4 Fig) leads to the separation of some artichoke genotypes originating from the Apulia region, Italy (Carciofo noscio locale, Nero di Ostuni, Nero di Castrignano, Spinoso di Galatina) which are all dark in colour, but with two different head shapes. In particular, Carciofo noscio locale and Nero di Ostuni have a cylindrical flower head, while the others show a very particular flower head with everted bracts. We have attributed the generic name of ˝Nero del Salento˝ (˝Black from Salento˝) to this last typology (https://biodiversitapuglia.it/carciofo-nero-del-salento-varieta-locale-tutto-particolare/), Salento being a geographical area in the south of Apulia region. To our best knowledge, this local variety is present only in Salento, where it is quite diffused, although it is mainly grown in family gardens for local consumption, and is difficult to find in stores or markets.

**Fig 2:**
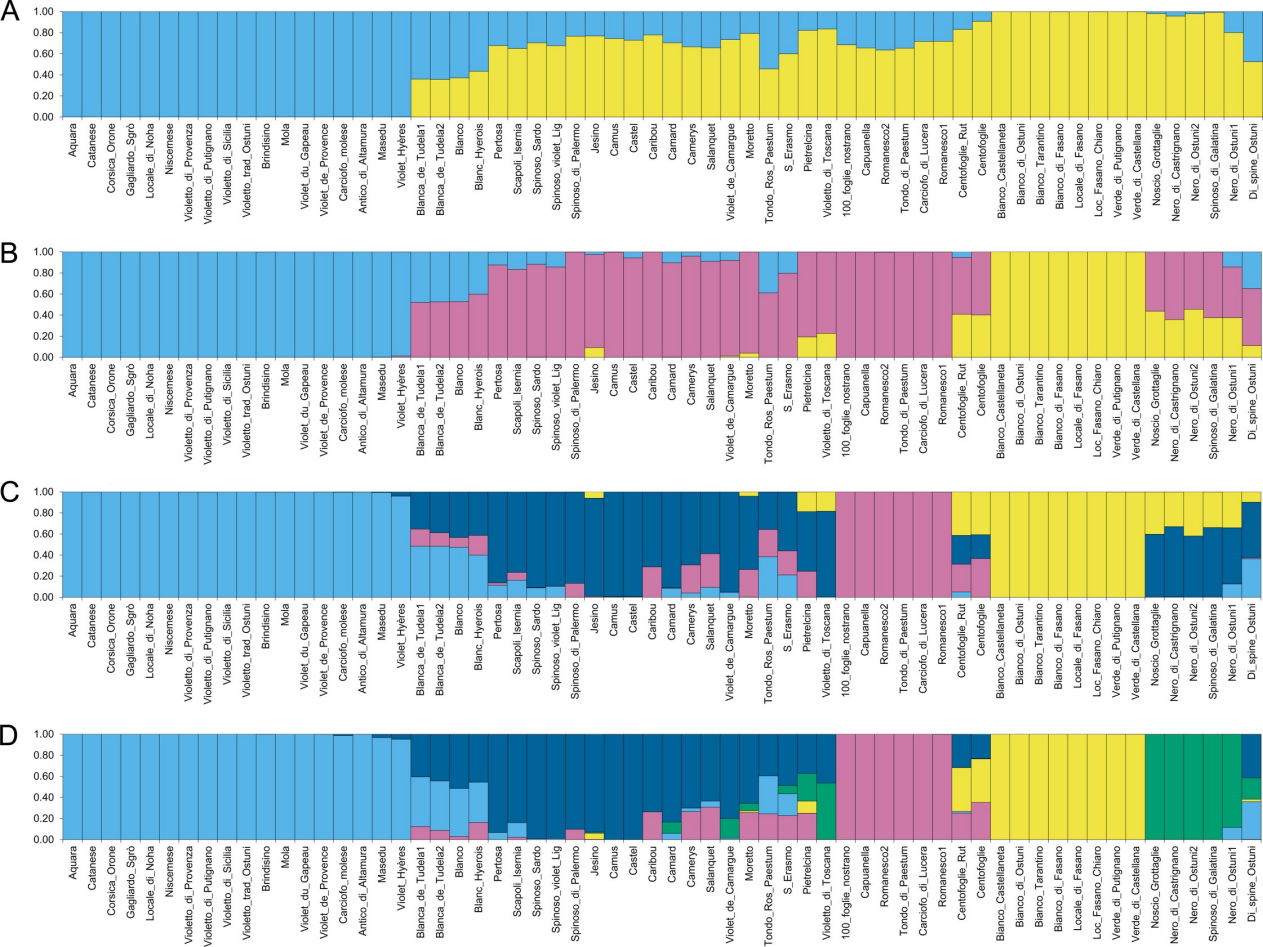
Structure analysis of globe artichoke genotypes. Clusters inferred from the population structure analysis at (A) K = 2, (B) K = 3 and (C) K = 4, (D) K = 5. Numbers on the y-axis show the subgroup membership. Genotype names are reported on the x axis. The colours of the bars indicate the groups identified through the STRUCTURE program.

In order to achieve a better picture of the globe artichoke genetic structure, the analysis was also run on a subset of the original samples containing only the globe artichoke genotypes. In this case, the best fitting model was represented again by K = 2 (Fig 2A), which separates the ˝Catanesi˝ artichokes on one side (light blue), and all the other typologies on the other side (yellow), with several admixed samples. The second and the third best K values were K = 3 and K = 4, respectively. At K = 3 (Fig 2B), the first group is composed of the ˝Catanesi˝ types (light blue). The second group (mainly dark pink) includes the ˝Romaneschi˝ *sensu lato* (except for Tondo Rosso di Paestum and the two Centofoglie genotypes, which are admixed), violet and spiny types. The two Blanca de Tudela genotypes, Blanc Hyerois and Blanco are admixed between the first two groups. The third group includes the ˝Green Apulian˝ artichokes (yellow). Some other local varieties are admixed between groups 2 and 3. At K = 4 (Fig 2C), the ˝Catanesi˝ (light blue) and the ˝Green Apulian˝ (yellow) groups of the previous clustering at K = 3 are generally maintained, while the other large K = 3 group is split into two smaller sets with a higher number of admixed samples. Of these, one (dark pink) includes the ˝Romaneschi˝ *sensu stricto* (Romanesco1, Romanesco2, Capuanella, Tondo di Paestum, Carciofo di Lucera, 100_foglie_nostrano), while the second group (dark blue) is composed of some other ˝Romaneschi˝ *sensu lato* genotypes (Jesino, Pertosa, Scapoli-Isernia, Camus, Castel, Camard), together with ˝Spinosi˝ types and Violetto di Toscana. The other two ˝Violetti˝, namely Moretto and S. Erasmo are admixed, with a membership coefficient for this group of 0.70 and 0.56, respectively. Several other admixed genotypes are observed. At K = 5 (Fig 2D), the group of dark artichokes from Apulia separates (green). In the K = 6 solution (S4B Fig), besides the previous groups, the ˝Romaneschi˝ types are further divided and form three subsets, one of which includes the two French Camus and Castel varieties (orange), while the other French ˝Romaneschi˝ are admixed (˝q˝ value ranging from 0.67 to 0.72). The second ˝Romaneschi˝ group (dark pink) is composed of the ˝Romaneschi˝ *sensu stricto*, while the third one (dark blue) includes two large-headed green genotypes (Pertosa and Scapoli_Isernia) together with ˝Spinosi˝ types. At K = 7 (S4B Fig), the two genotypes of Blanca de Tudela and Blanc Hyerois, which were previously admixed, form a separate group together with Blanco (dark orange).

Concerning the cardoon dataset, the most probable model was obtained for K = 2 (S3 Fig), where the two groups correspond to cultivated and wild cardoons. The leafy cardoon group also includes the wild cardoons from the Iberian Peninsula. Two genotypes of wild cardoon from Tunisia, and one from Sicily are admixed, with a higher ˝q˝ value for the wild group. Increasing the number of K, the wild cardoon group splits into smaller clusters according to the geographical origin of the samples, while the cultivated cardoons remain as a single group, and the number of admixed samples increases (S5 Fig). It is interesting to notice that, at K = 8, the second most probable K (S3 Fig), wild genotypes from the different countries are separated from one another, although each geographical area is represented by a limited number of genotypes. Among the Italian wild cardoons, a regional subdivision can be observed, with genotypes from Apulia and Basilicata placed in the same group, samples from Sardinia and Latium in another small group, and genotypes from Sicily and Calabria sharing admixed ancestry. Moreover, the wild cardoon from Portugal and one of the wild cardoons from Spain separate from the group of cultivated cardoons.

In *C*. *cardunculus*, the expected heterozygosity obtained within STRUCTURE clusters at K = 2 revealed a lower value in cardoons (0.160), compared to the globe artichoke, which showed an expected heterozygosity of 0.216. Allele frequency divergence, or ˝Net nucleotide distance˝ between clusters was obtained for the whole dataset at K = 5, where wild and cultivated cardoons were separated, and the globe artichoke samples formed three groups. Net represents the average of pairwise difference between alleles from different groups, excluding the amount of variation located within each group. Similar groups have distances approaching to 0. In our study, the lowest divergence (0.086) was observed between wild and cultivated cardoons, while the highest Net was detected between two groups of artichokes, ˝Catanesi˝ and ˝Green Apulian˝ and between ˝Catanesi˝ and wild cardoons, with similar values, 0.202 and 0.201, respectively.

### Genetic relationships

**Fig 3:**
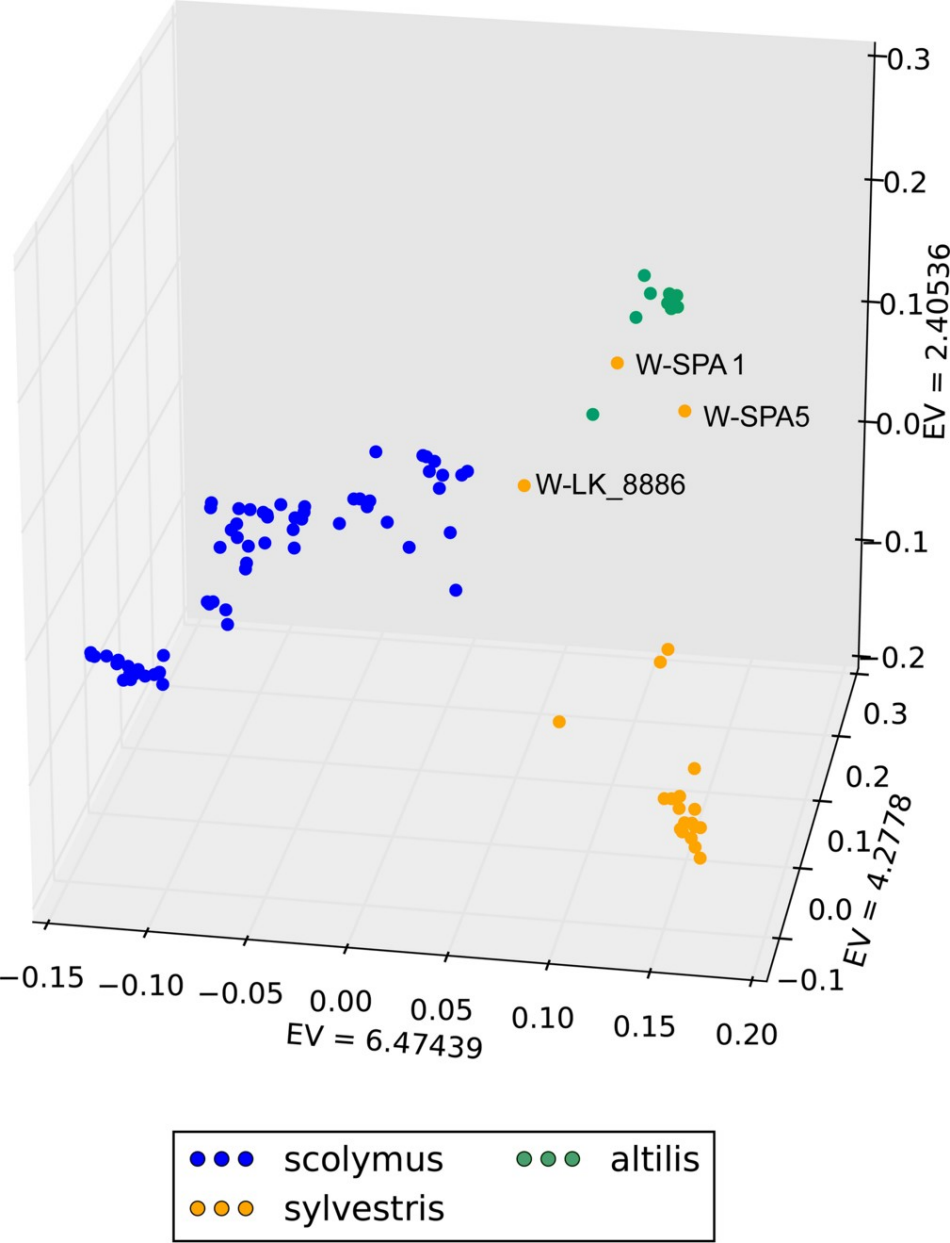
Graph of the first three axes from a Principal component analysis (PCA) of the 92 *Cynara cardunculus* genotypes analysed. EV: eigenvalue. Blue: globe artichoke; green: cultivated cardoon; orange: wild cardoon.

**Fig 4:**
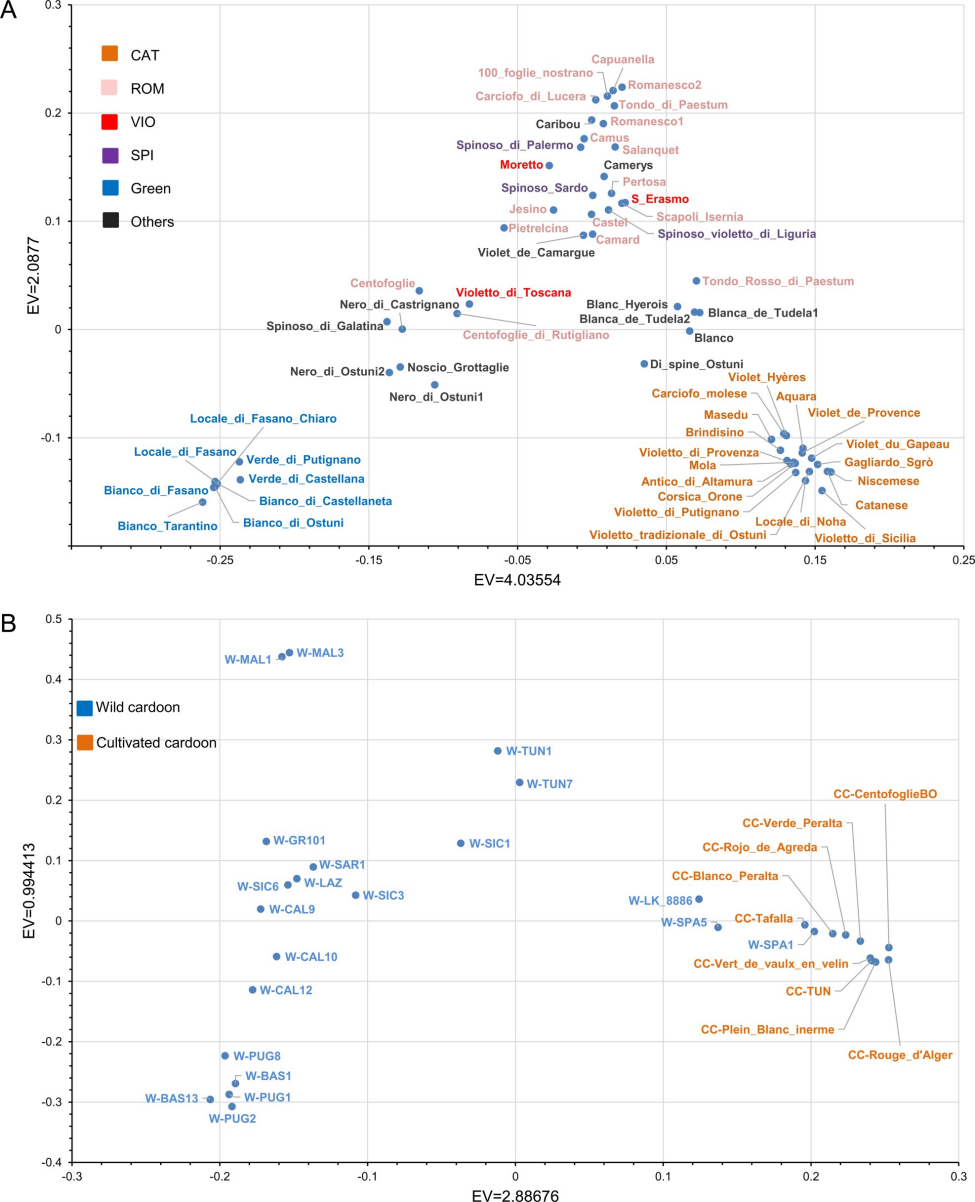
Graph of the first two axes from a Principal component analysis (PCA) of (A) 62 globe artichoke and (B) 30 wild and cultivated cardoon genotypes. EV: eigenvalue. Colors indicate globe artichoke types (A), or wild or cultivated cardoon (B). CAT: Catanesi: ROM: Romaneschi; VIO: Violetti; SPI: Spinosi; Green: Green Apulian artichokes; Others: other types.

In order to assess genetic relationships among *C*. *cardunculus* genotypes, PCA and Neighbour-Joining clustering were performed. For both analyses, three datasets, referring to i) the complete *C*. *cardunculus* collection, ii) artichokes, and iii) cardoons, were used separately. In the 3D PCA plot for the whole dataset (Fig 3), wild cardoon, cultivated cardoon and globe artichoke samples are differently positioned. Globe artichoke samples (blue) form a quite dispersed cloud. Some samples, namely the wild cardoon from Portugal and the two wild cardoons from Spain, appear quite distant from the other genotypes belonging to the same taxonomic entity. In particular, the latter ones are closer to the leafy cardoons than to the other wild cardoons, while the wild cardoon from Portugal is placed between cultivated cardoons and globe artichokes. The eastern wild material shows a quite sparse distribution, indicating a degree of differentiation among genotypes originating from various geographical areas. The PCA plot restricted to the globe artichoke germplasm (Fig 4A) shows two main distinct groups: the ˝Catanesi˝ (bottom right), and the ˝Green Apulian˝ (bottom left) artichokes. The remaining genotypes are scattered in the central-upper part of the plot, and no clear clustering can be observed for them based on the variety, except for some of the ˝Romaneschi˝ types in the upper part. The PCA for the cardoons (Fig 4B) separates the quite compact group of cultivated cardoons (bottom right) from wild (left) cardoons. Exceptions are represented by wild cardoons from the Iberian Peninsula, placed together with the cultivated cardoons. The eastern wild cardoons are spread in the left part of the graph, indicating a quite high differentiation of this group originating from three European countries and Tunisia.

**Fig 5:**
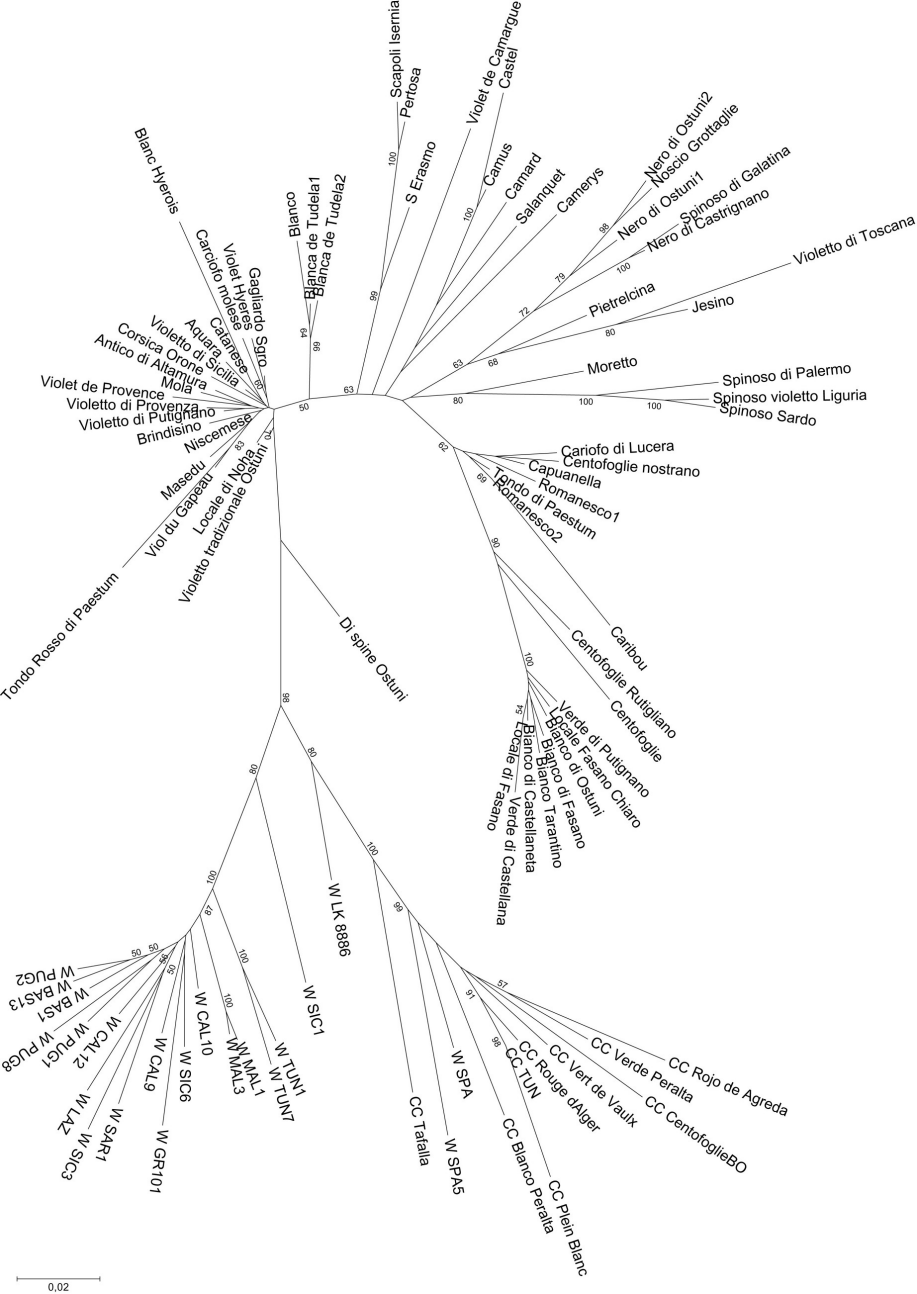
Neighbor-Joining tree obtained from SNP data on the whole dataset of *Cynara cardunculus* genotypes Number on tree branches indicate bootstrap values (≥ 50).

**Fig 6:**
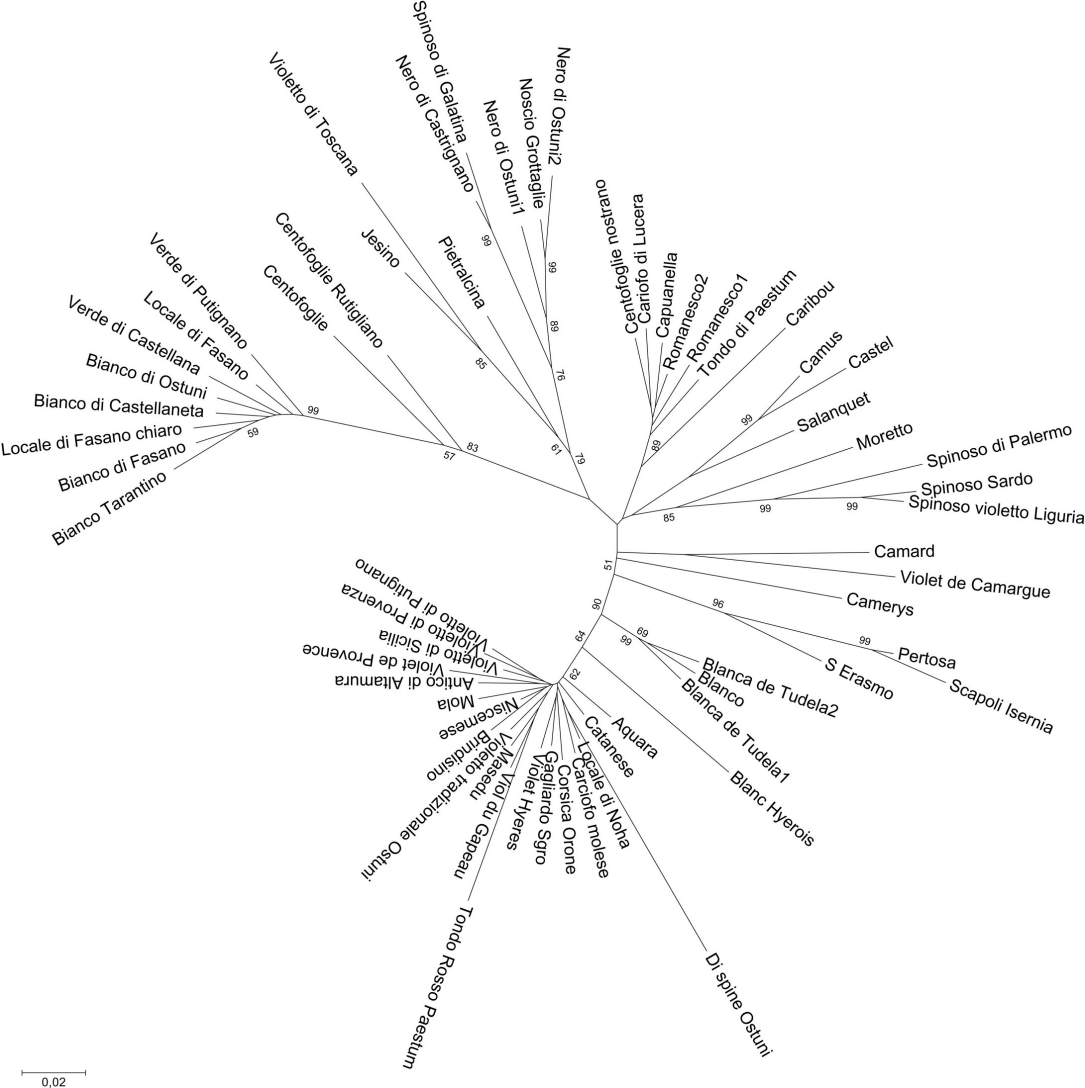
Neighbor-Joining tree obtained from SNP data on the globe artichoke genotypes. Number on tree branches indicate bootstrap values (≥ 50).

The Neighbor-Joining tree obtained from the whole dataset (Fig 5) highlights two main branches, one including the globe artichokes, and the other the rest of the material. This second branch is subdivided into two forks: wild cardoons on one side and cultivated cardoons on the other, this latter group also comprising the wild cardoons from Spain and Portugal. The landrace Di spine Ostuni is placed between the cardoon and the artichoke groups, thus substantiating the idea that this is a hybrid type. Within the artichoke cluster, subgroups can be identified, three of which are compact, i.e. the ˝Catanesi˝, the ˝Green Apulian˝, and some of the ˝Romaneschi˝ types (Romanesco1, Romanesco2, Capuanella, 100_Foglie_Nostrano, Tondo di Paestum, Carciofo_di_Lucera, and Caribou). The admixed ˝Tondo Rosso di Paestum˝ artichoke is included in the ˝Catanesi˝ group, although at a high distance. The other genotypes are quite scattered in the artichoke branch, even though small subsets can be recognised: the Apulian dark-headed artichokes (Noscio Grottaglie, Nero di Ostuni, Spinoso di Galatina, Nero di Castrignano), the ˝Spinosi˝ types (Spinoso di Palermo, Spinoso Violetto di Liguria, Spinoso Sardo). The two Blanca de Tudela genotypes, together with Blanco, are positioned between ˝Catanesi˝ and the other artichoke types. When performing the clustering analysis only with the globe artichoke dataset (Fig 6), the main groups observed are again ˝Catanesi˝ and smaller clusters, such as the ˝Green Apulian˝, some of the ˝Romaneschi˝, ˝Spinosi˝, and the previously identified Apulian dark-headed artichokes. The phylogenetic tree constructed for the cardoons (S6 Fig) is divided into two clusters: the wild and the cultivated cardoons. Also in this case, the Iberian wild cardoons are included in the group of cultivated cardoons.

### LD decay

**Fig 7:**
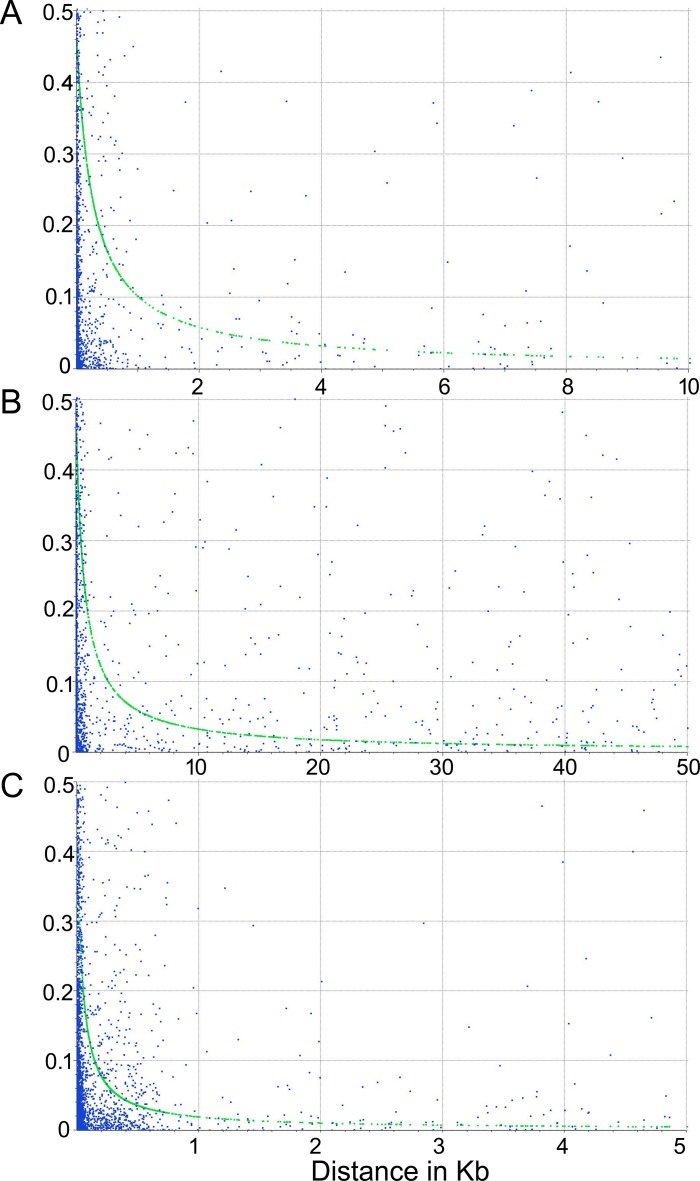
LD decay for (A) the whole dataset of *Cynara cardunculus* genotypes, (B) the globe artichoke subset, (C) cardoon samples. LD decay determined by squared correlations of allele frequencies (r^2^) against distance between polymorphic sites.

To assess the extent of LD decay, the estimate of r^2^ for all pairs of SNP loci linked on the same genome scaffold, was calculated. The LD decay was evaluated for the whole dataset (including wild and cultivated material), for the globe artichoke group, and for the cardoon collection separately. The LD decay observed for the whole *C*. *cardunculus* array was quite rapid, with r^2^ = 0.2 after 0.35 kb (Fig 7). When the analysis was carried out on the two subsets of the germplasm collection, a different behaviour was observed, as LD decay in globe artichokes (r^2^ = 0.2 after 0.92 kb) was considerably slower compared to that of cardoons (r^2^ = 0.2 after 0.05kb).

## Discussion

Genotyping by sequencing is a high-throughput and cost-effective technology to detect and genotype a large number of polymorphisms at the genome scale. By surveying genotypes belonging to the three taxa of *C*. *cardunculus* species, we provide the first wide SNP genotyping study for germplasm of this species complex, as a previous investigation based on RAD sequencing was focused on parents of mapping populations [44]. GBS performed with rare-cutter enzymes, such as *Pst*I used in this study, are generally useful to increase sequence depth [45]. This fits with the need of our study, requiring high depth for efficiently calling heterozygous genotypes, frequently occurring in an in outcrossing species such as *C*. *cardunculus*, at multiplexed level. The number of tags mapped on the globe artichoke genome was not high, and this might be due to the draft-quality of the reference genome available, which was organized in scaffolds. However, even applying severe filtering parameters (mostly MAF>0.05 and p-value for HWE>10^−6^), we detected thousands of SNP polymorphisms for downstream analyses.

Analysis of SNP data was performed on three separate datasets: i) the whole *C*. *cardunculus* collection; ii) globe artichoke samples; iii) wild and leafy cardoon samples. STRUCTURE analysis provided K = 2 as the most probable number of populations for the whole dataset, splitting the cardoons (wild and cultivated) from the globe artichokes. For K values from 2 to 4, cultivated cardoon grouped together with wild cardoon, suggesting that cultivated cardoons retain a higher proportion of wild alleles. According to Dempewolf et al. [46], both *Cynara* crops can be regarded as semi-domesticated plants; indeed globe artichoke and leafy cardoon derive from two domestication events leading to larger flower heads or leaf stalks, respectively [11]. However, differently from globe artichoke, leafy cardoon shows the persistence of ancient wild traits, related to domestication, such as a higher number of plant branches, small capitula, etc. [11, 21].

Based on the present SNP analysis, the cultivated cardoon cluster also includes wild cardoon genotypes collected in Spain, corroborating the idea that this material might indeed represent a feral form of leafy cardoons [21]. This is also supported by phenotypic traits of these plants, which resemble cultivated cardoons as they show few or no thorns, large and tall size, and a habit and morphology different from that of other wild material (Sonnante, personal observations).

At K = 5, the highest divergence among groups is observed between the ˝Catanesi˝ and the ˝Green Apulian˝ artichoke and between the ˝Catanesi˝ and the wild material. The separation of the ˝Catanesi˝ group is also highlighted by Neighbor-Joining hierarchical clustering and PCA analysis. ˝Catanesi˝ possess a typical trait, in common with a few other varieties (e.g. Blanca de Tudela), that is earliness or re-flowering (they bloom in autumn and in spring). This important agronomic trait might have been selected in more recent times, leading to a sharp separation of ˝Catanesi˝ from the other artichokes and from the wild cardoons, which are also late-flowering plants since they bloom in spring. Additionally, the high homogeneity observed within the ˝Catanesi˝ group suggests that these artichokes may derive from the same genetic material, which probably, as the name indicates (from Catania, a town in Sicily), originated in Sicily. Later on, this variety possibly diffused, especially in the Apulia region (Italy) and in other areas (e.g. France), where it has given rise to a number of similar local varieties (e.g. Brindisino, Mola, Violet de Provence, etc.).

The ˝Romaneschi˝ types appear quite a heterogeneous group, and this can be perceived from the results of STRUCTURE, NJ clustering and PCA analyses. Only some of the ˝Romaneschi˝ genotypes are grouped together, while the others are scattered. This output is consistent with previous results obtained using different molecular markers [21, 24, 47] and suggests that the artichoke morpho-groups are not always substantiated by the genetic composition and relationships between genotypes. The difficulty in recognizing a precise definition for the four traditional morpho-groups, apart from the ˝Catanesi˝ and a few ˝Romaneschi˝, might suggest that just a few genes control those morphological traits determining the differences among groups. Especially for ˝Romaneschi˝ types, the circular or large transverse elliptical forms as described in the UPOV (International Union for the Protection of New Varieties of Plants) descriptors for artichoke (http://www.upov.int/edocs/tgdocs/en/tg184.pdf), do not identify a homogeneous group (usually called ˝Romaneschi˝), but diverse artichokes with a variable genetic background.

Blanca de Tudela is a traditional widespread Spanish artichoke variety; it is green-coloured and, similarly to the ˝Catanesi˝ artichokes, early flowering. Following STRUCTURE analysis, Blanca de Tudela genotypes appear admixed between the ˝Catanesi˝ types and other typologies. Accordingly, in the NJ tree as well as in the PCA plot, this artichoke type is placed between ˝Catanesi˝ and the other artichokes. With both dominant and SSR markers, Blanca de Tudela artichoke had already shown this intermediate position [21, 47]. Therefore, we suppose that Blanca de Tudela might have derived from a cross between a ˝Catanesi˝ type artichoke with another typology.

The ˝Green Apulian˝ genotypes represent a compact group that is quite distinguishable from the other globe artichokes. To our best knowledge, these artichokes, as well as the ˝Nero del Salento˝ typology (see above) are grown just in the Apulia region (Southern Italy). It has been noted that Southern Italy, and especially the Apulia region, represents a centre of diversity for many crops, where still traditional farming systems can be found and ancient and diversified crops are cultivated [8, 9, 12, 48, 49].

The SNP analyses highlighted that globe artichoke retains a higher level of heterozygosity compared to wild and cultivated cardoon. This result is in agreement with previous studies carried out with SSR markers [12, 21, 47] and with a recent genome resequencing study [29]. Clonally propagated crops are generally outcrossing plants, and inbred individuals usually show lower vigour deriving from inbreeding depression [50]. It is possible that highly heterozygous globe artichoke genotypes were selected to maximize heterotic effects, which have been then maintained by farmers by clonal propagation (indeed, all the globe artichoke germplasm analysed in this study is clonally propagated). In fact, sexual recombination can knock down advantageous genetic combinations, while clonal multiplication preserves them [51]. Another, not mutually exclusive, hypothesis for high heterozygosity levels is that clonal lineages are likely to accumulate slightly deleterious mutations, differently from sexually propagated cardoons in which such mutational events are eliminated by recombination or selection.

Linkage disequilibrium is the non-random association of alleles at different loci, and is influenced by various factors. For instance, domestication, population subdivision, selection can enhance LD in the genome [52]. Generally, LD decays faster in outcrossing species than in self-fertilizing plants, but outcrossing vegetatively propagated plants are an exception [53]. The difference in LD decay between the globe artichoke group and the cardoon cluster might be related to the different reproductive and propagation system of the taxa within *C*. *cardunculus*, and to domestication and selective pressure preserving specific haplotype blocks. Although all three taxa are outcrossing, the clonally propagated globe artichoke shows a slower LD decay compared to wild and cultivated cardoon, probably due to the few recombination events happened during its long breeding cycle. A relatively slow LD decay has been observed in other perennial or clonally propagated crops, such as sugarcane and potato [54, 55]. Moreover, domestication events and selective pressure on improved material have been shown to decrease LD decay passing from wild relatives to landraces to modern cultivars in some species such as soybean [56], and in the development of elite populations in maize [57] and barley [58].

## Conclusion

The analysis of SNPs derived from GBS technology in *C*. *cardunculus* highlighted a strong structure separating the globe artichoke from the cardoon material, both wild and cultivated. The wild material from Spain was included in the cultivated cardoon group, supporting the idea that this might represent a feral form. Other wild genotypes might have derived from hybridization between wild and cultivated material. Structures were also observed within the globe artichoke collection, with genotypes belonging to the ˝Catanesi˝ or ˝Green Apulian˝ types forming well structured groups. Moreover, the late flowering large round-shaped ˝Romaneschi˝ type cannot be considered as a single, genetically uniform group. The globe artichokes displayed a slower LD decay compared to cardoons possibly due to the different reproductive system and to domestication and selective pressure.

## Supporting information

S1 Table List of the material analysed. All ˝varieties/ecotypes˝ tagged with ˝CC˝ represent cultivated cardoons, the ones tagged with ˝W˝ indicate wild cardoons, and the ones not tagged in this way are the globe artichokes. Within globe artichokes, CAT: ˝Catanesi˝; VIO: ˝Violetti˝; SPI: ˝Spinosi˝; ROM: ˝Romaneschi˝; OFF: off types;?: uncertain attribution. In red, artichoke samples not used for diversity and STRUCTURE analyses.
(DOCX)

S2 Table Genomic position and ID number of the *Cynara cardunculus* SNPs after filtering. Each worksheet contains the SNPs for a single dataset: 1. Total *C*. *cardunculus*; 2. Globe artichoke; 3. Cardoon.
(XLSX)

S1 Fig Bar charts describing the proportion of SNP heterozygous distribution in C. cardunculus before (A) and after (B) HWE filtering (p-value >10−6)
(PDF)

S2 Fig Pie chart showing the frequency of substitution types of the identified SNPs. (PDF)

S3 Fig Determination of the most probable K by means of ΔK statistics. The number of subpopulations (K) was identified based on maximum likelihood and ΔK values. (A) Whole dataset; (B) Globe artichoke dataset; (C) Cardoon dataset.
(PDF)

S4 Fig STRUCTURE analysis at K = 6 and K = 7. (A): complete *C*. *cardunculus* dataset; (B): globe artichoke dataset. Numbers on the y-axis show the subgroup membership. Genotype names are reported on the x axis. The colours of the bars indicate the groups identified through the STRUCTURE program.
(TIF)

S5 Fig STRUCTURE analysis of cardoon dataset. Numbers on the y-axis show the subgroup membership. Genotype names are reported on the x axis. The colours of the bars indicate the groups identified through the STRUCTURE program.
(PDF)

S6 Fig Neighbor-Joining tree obtained from SNP data on the wild and cultivated cardoon collection. Number on tree branches indicate bootstrap values (≥ 50).
(TIF)

## Abbreviations

GBS: Genotyping by sequencing
MCMC: Markov Chain Monte Carlo
LD: linkage disequilibrium
SVS: SNP & Variation Suite
MAF: Minor allele frequency
PCA: Principal component analysis
EM: Expectation-maximization
F_ST_: Fixation index
UPOV: International Union for the Protection of New Varieties of Plants
