# Supplementary material for: Genotyping-by-sequencing highlights patterns of genetic structure and domestication in artichoke and cardoon
Source: PLoS One. 2018 Oct 23;13(10):e0205988. doi: 10.1371/journal.pone.0205988 (PMC6198968; doi:10.1371/journal.pone.0205988)
Supplement: S1 Table — All ˝varieties/ecotypes˝ tagged with ˝CC˝ represent cultivated cardoons, the ones tagged with ˝W˝ indicate wild cardoons, and the ones not tagged in this way are the globe artichokes. Within globe artichokes, CAT: ˝Catanesi˝; VIO: ˝Violetti˝; SPI: ˝Spinosi˝; ROM: ˝Romaneschi˝; OFF: off types;?: uncertain attribution. In red, artichoke samples not used for diversity and STRUCTURE analyses. (DOCX) [file pone.0205988.s001.docx]

**S1 Table**. List of the material analysed. All “varieties/ecotypes” tagged with “CC” represent cultivated cardoons, the ones tagged with “W” indicate wild cardoons, and the ones not tagged in this way are the globe artichokes. Within globe artichokes, CAT: “Catanesi”; VIO: “Violetti”; SPI: “Spinosi”; ROM: “Romaneschi”; OFF: off types; ?: uncertain attribution. In red, artichoke samples not used for diversity and STRUCTURE analyses.

| **No** | **Variety/ecotype** | **Tipology/Taxon** | **Origin** |  | **No** | **Variety/ecotype** | **Tipology/Taxon** | **Origin** |
| --- | --- | --- | --- | --- | --- | --- | --- | --- |
| 1 | Carciofo_molese | CAT | Italy |  | 49 | Nero_di_Ostuni1 | OFF | Italy |
| 2 | Mola | CAT | Italy |  | 50 | Nero_di_Ostuni2 | OFF | Italy |
| 3 | Brindisino | CAT | Italy |  | 51 | Nero_di_Castrignano | OFF | Italy |
| 4 | Catanese | CAT | Italy |  | 52 | Blanco | ? | Spain |
| 5 | Gagliardo_Sgrò | CAT | Italy |  | 53 | Noscio_Grottaglie | ? | Italy |
| 6 | Niscemese | CAT | Italy |  | 54 | Bianco_di_Ostuni | OFF | Italy |
| 7 | Violetto_di_Sicilia | CAT | Italy |  | 55 | Verde_di_Castellana | OFF | Italy |
| 8 | Violetto_di_Provenza | CAT | Italy |  | 56 | Bianco_Tarantino | OFF | Italy |
| 9 | Violet_de_Provence | CAT | France |  | 57 | Verde_di_Putignano | OFF | Italy |
| 10 | Masedu | CAT | Italy |  | 58 | Bianco_di_Fasano | OFF | Italy |
| 11 | Violet_Hyères | CAT | France |  | 59 | Locale_di_Fasano_Chiaro | OFF | Italy |
| 12 | Violet_du_Gapeau | CAT | France |  | 60 | Bianco_di_Castellaneta | OFF | Italy |
| 13 | Violetto_tradizionale_di_Ostuni | CAT | Italy |  | 61 | Locale_di_Fasano | OFF | Italy |
| 14 | Locale_di_Noha | CAT | Italy |  | 62 | Di_spine_Ostuni | OFF | Italy |
| 15 | Antico_di_Altamura | CAT | Italy |  | 63 | CC-Plein_Blanc_inerme | CC | France |
| 16 | Aquara | CAT | Italy |  | 64 | CC-Vert_de_vaulx_en_velin | CC | France |
| 17 | Corsica_Orone | ? | France |  | 65 | CC-Rouge_dAlger | CC | France |
| 18 | Blanca_de_Tudela1 | OFF | Spain |  | 66 | CC-Blanco_Peralta | CC | Spain |
| 19 | Blanca_de_Tudela2 | OFF | Spain |  | 67 | CC-Verde_Peralta | CC | Spain |
| 20 | Romanesco1 | ROM | Italy |  | 68 | CC-Rojo_de_Agreda | CC | Spain |
| 21 | Romanesco2 | ROM | Italy |  | 69 | CC-Tafalla | CC | Spain |
| 22 | Capuanella | ROM | Italy |  | 70 | CC-CentofoglieBO | CC | Italy |
| 23 | Tondo_di_Paestum | ROM | Italy |  | 71 | CC-TUN | CC | Tunisia |
| 24 | Tondo_Rosso_di_Paestum | ROM | Italy |  | 72 | W-SPA1 | W | Spain |
| 25 | 100_foglie_nostrano | ROM | Italy |  | 73 | W-SPA5 | W | Spain |
| 26 | Centofoglie_di_Rutigliano | ROM | Italy |  | 74 | W-LK_886 | W | Portugal |
| 27 | Centofoglie | ROM | Italy |  | 75 | W-TUN1 | W | Tunisia |
| 28 | Pietrelcina | ROM | Italy |  | 76 | W-TUN7 | W | Tunisia |
| 29 | Carciofo_di_Lucera | ROM | Italy |  | 77 | W-MAL3 | W | Malta |
| 30 | Jesino | ROM | Italy |  | 78 | W-MAL1 | W | Malta |
| 31 | Pertosa | ROM | Italy |  | 79 | W-GR101 | W | Greece |
| 32 | Scapoli_Isernia | ROM | Italy |  | 80 | W-SIC3 | W | Italy |
| 33 | Camus | ROM | France |  | 81 | W-SIC1 | W | Italy |
| 34 | Camard | ROM | France |  | 82 | W-SIC6 | W | Italy |
| 35 | Castel | ROM | France |  | 83 | W-CAL12 | W | Italy |
| 36 | Salanquet | ROM | France |  | 84 | W-CAL10 | W | Italy |
| 37 | Camerys | ? | France |  | 85 | W-CAL9 | W | Italy |
| 38 | Caribou | ? | France |  | 86 | W-BAS13 | W | Italy |
| 39 | Blanc_Hyerois | ? | France |  | 87 | W-BAS1 | W | Italy |
| 40 | Violet_de_Camargue | ? | France |  | 88 | W-PUG8 | W | Italy |
| 41 | Violetto_di_Toscana | VIO | Italy |  | 89 | W-PUG2 | W | Italy |
| 42 | S_Erasmo | VIO | Italy |  | 90 | W-PUG1 | W | Italy |
| 43 | Moretto | VIO | Italy |  | 91 | W-LAZ | W | Italy |
| 44 | Violetto_di_Putignano | CAT | Italy |  | 92 | W-SAR1 | W | Italy |
| 45 | Spinoso_Sardo | SPI | Italy |  | 93 | Montelupone | ROM | Italy |
| 46 | Spinoso_di_Palermo | SPI | Italy |  | 94 | Violetto_di_Maremma | VIO | Italy |
| 47 | Spinoso_violetto_di_Liguria | SPI | Italy |  | 95 | Nero di Corigliano | OFF | Italy |
| 48 | Spinoso_di_Galatina | ? | Italy |  |  |  |  |  |
