# Supplementary figures and images for: Genotyping-by-sequencing highlights patterns of genetic structure and domestication in artichoke and cardoon
Source: PLoS One. 2018 Oct 23;13(10):e0205988. doi: 10.1371/journal.pone.0205988 (PMC6198968; doi:10.1371/journal.pone.0205988)

A

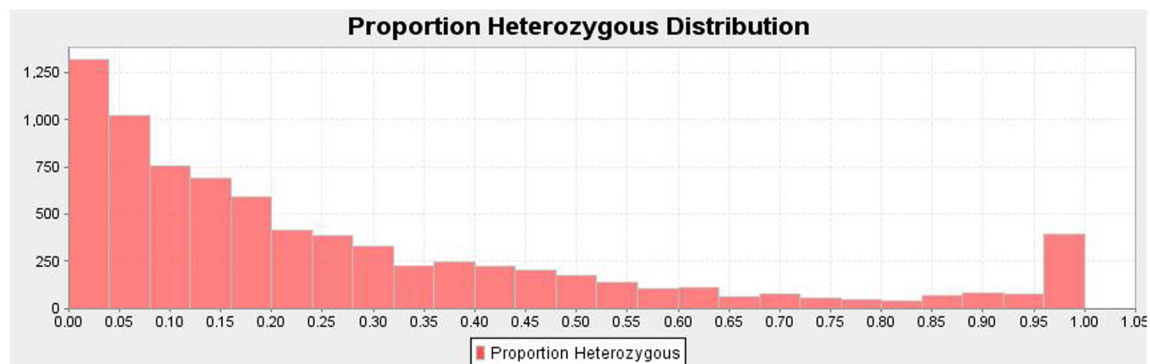

B

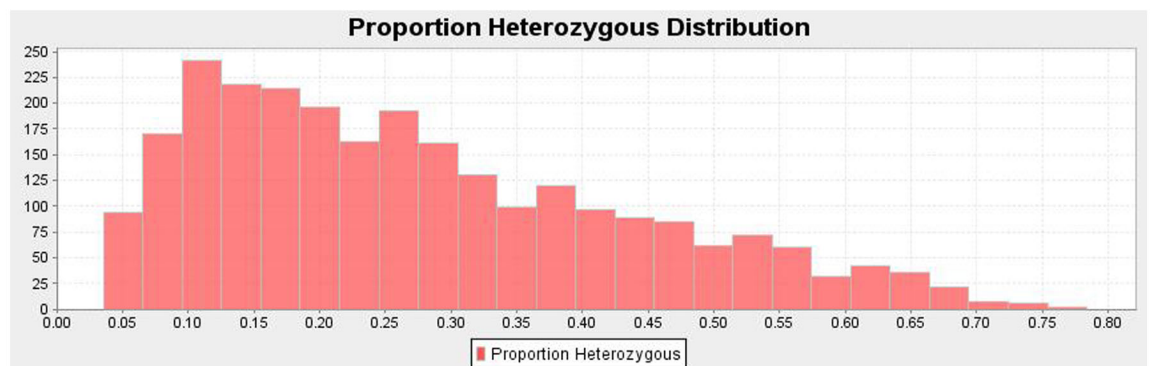

Supplement: S1 Fig — Bar charts describing the proportion of SNP heterozygous distribution in C. cardunculus before (A) and after (B) HWE filtering (p-value >10−6) (PDF) [file pone.0205988.s003.pdf]

# Transition/Transversion

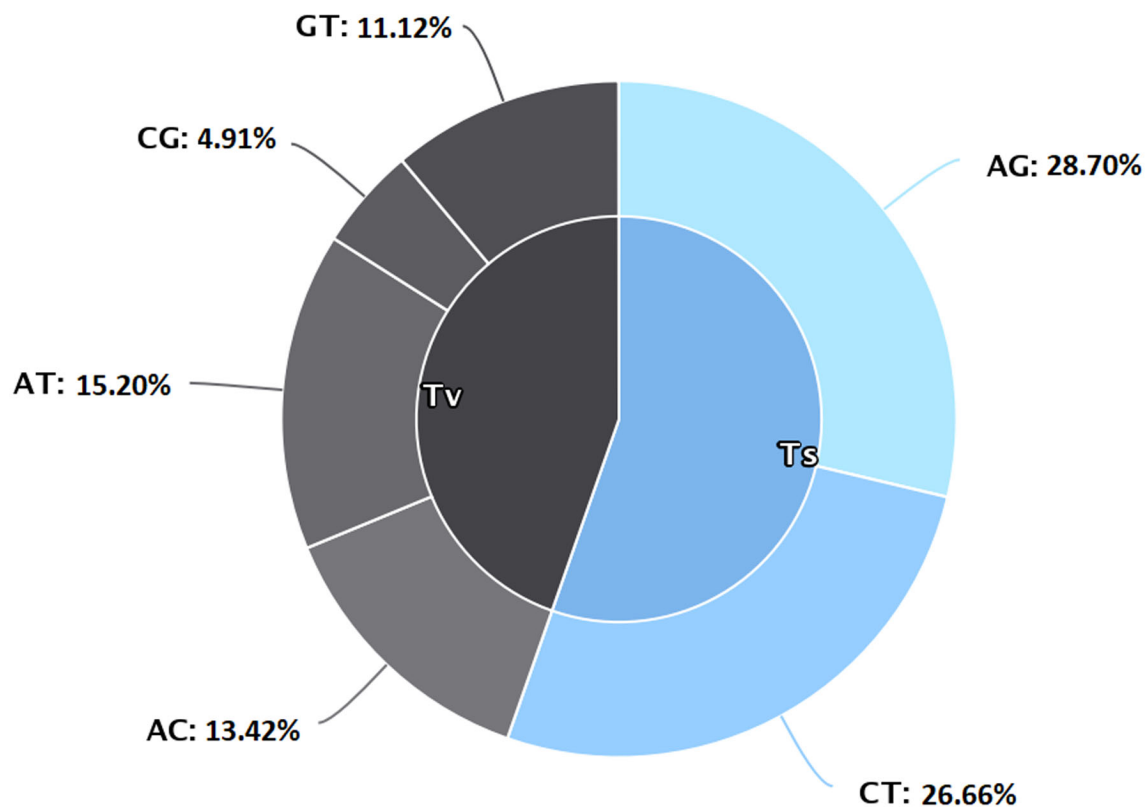

Supplement: S2 Fig — (PDF) [file pone.0205988.s004.pdf]

A

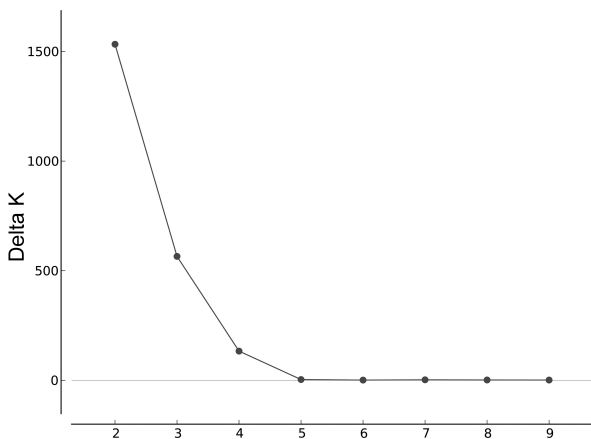

B

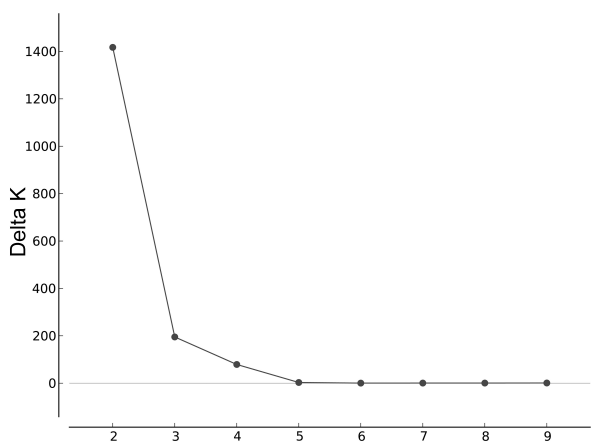

C

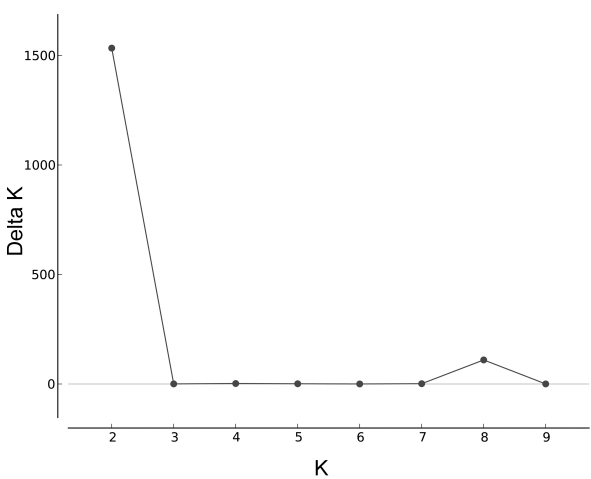

Supplement: S3 Fig — The number of subpopulations (K) was identified based on maximum likelihood and ΔK values. (A) Whole dataset; (B) Globe artichoke dataset; (C) Cardoon dataset. (PDF) [file pone.0205988.s005.pdf]

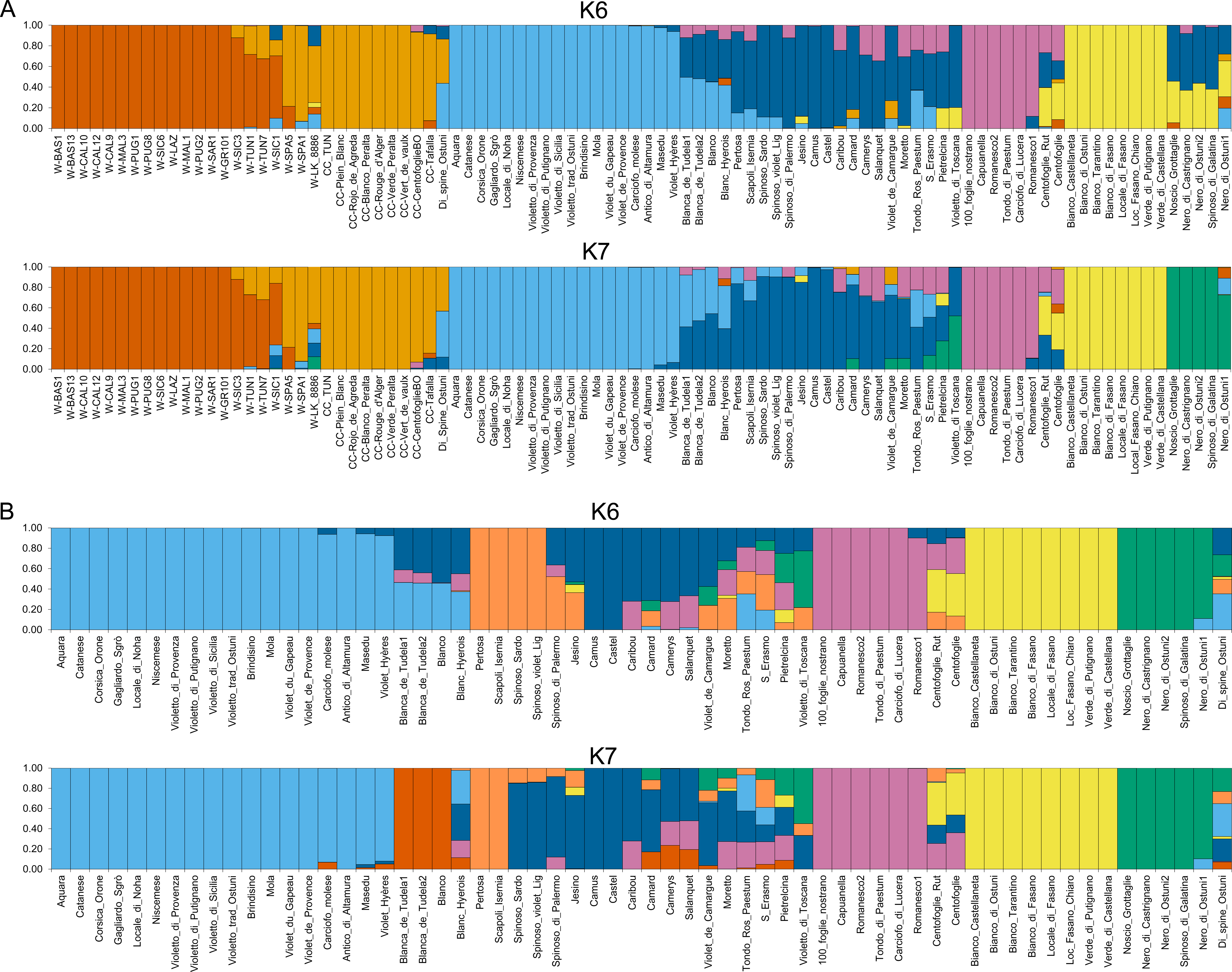

Supplement: S4 Fig — (A): complete C. cardunculus dataset; (B): globe artichoke dataset. Numbers on the y-axis show the subgroup membership. Genotype names are reported on the x axis. The colours of the bars indicate the groups identified through the STRUCTURE program. (TIF) [file pone.0205988.s006.tif]

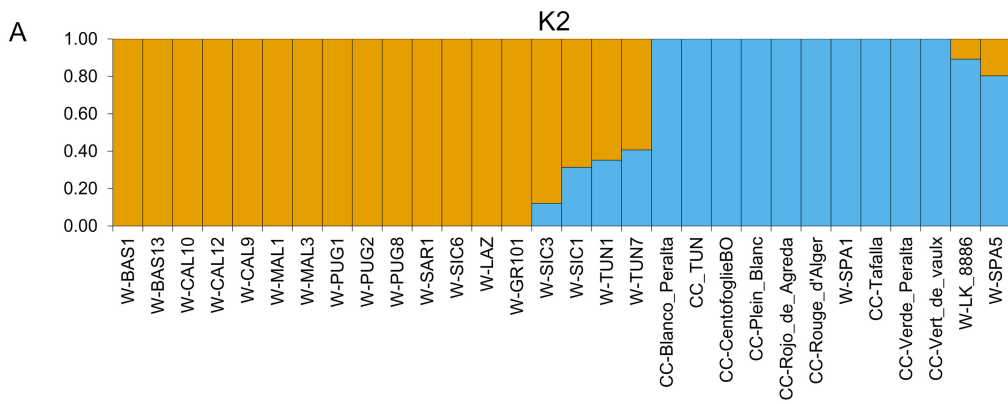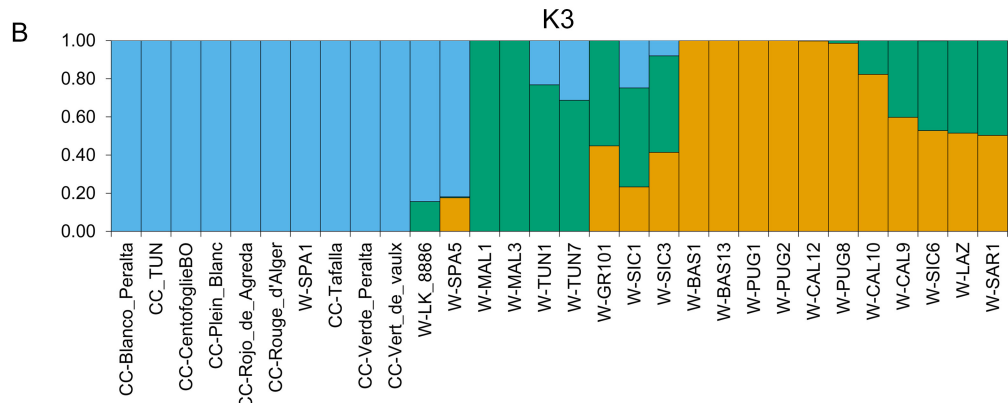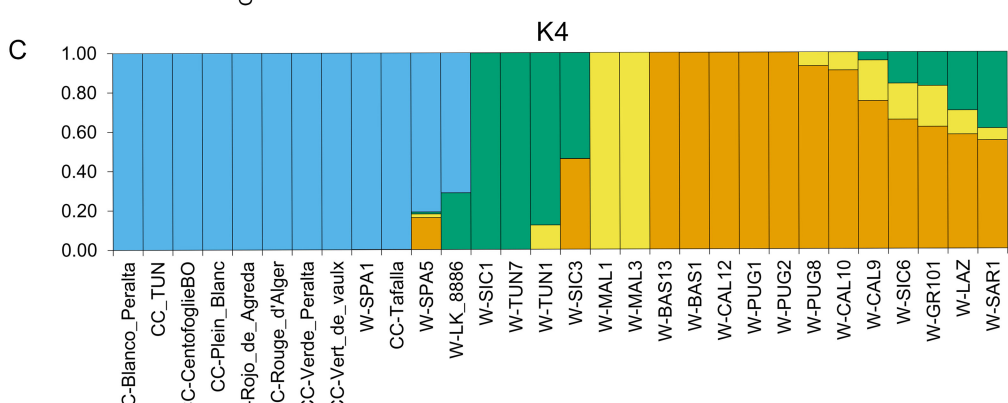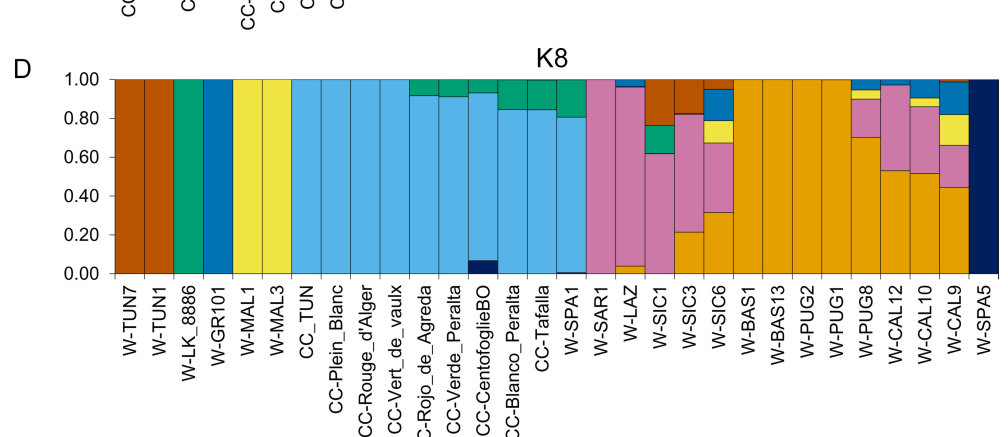

Supplement: S5 Fig — Numbers on the y-axis show the subgroup membership. Genotype names are reported on the x axis. The colours of the bars indicate the groups identified through the STRUCTURE program. (PDF) [file pone.0205988.s007.pdf]

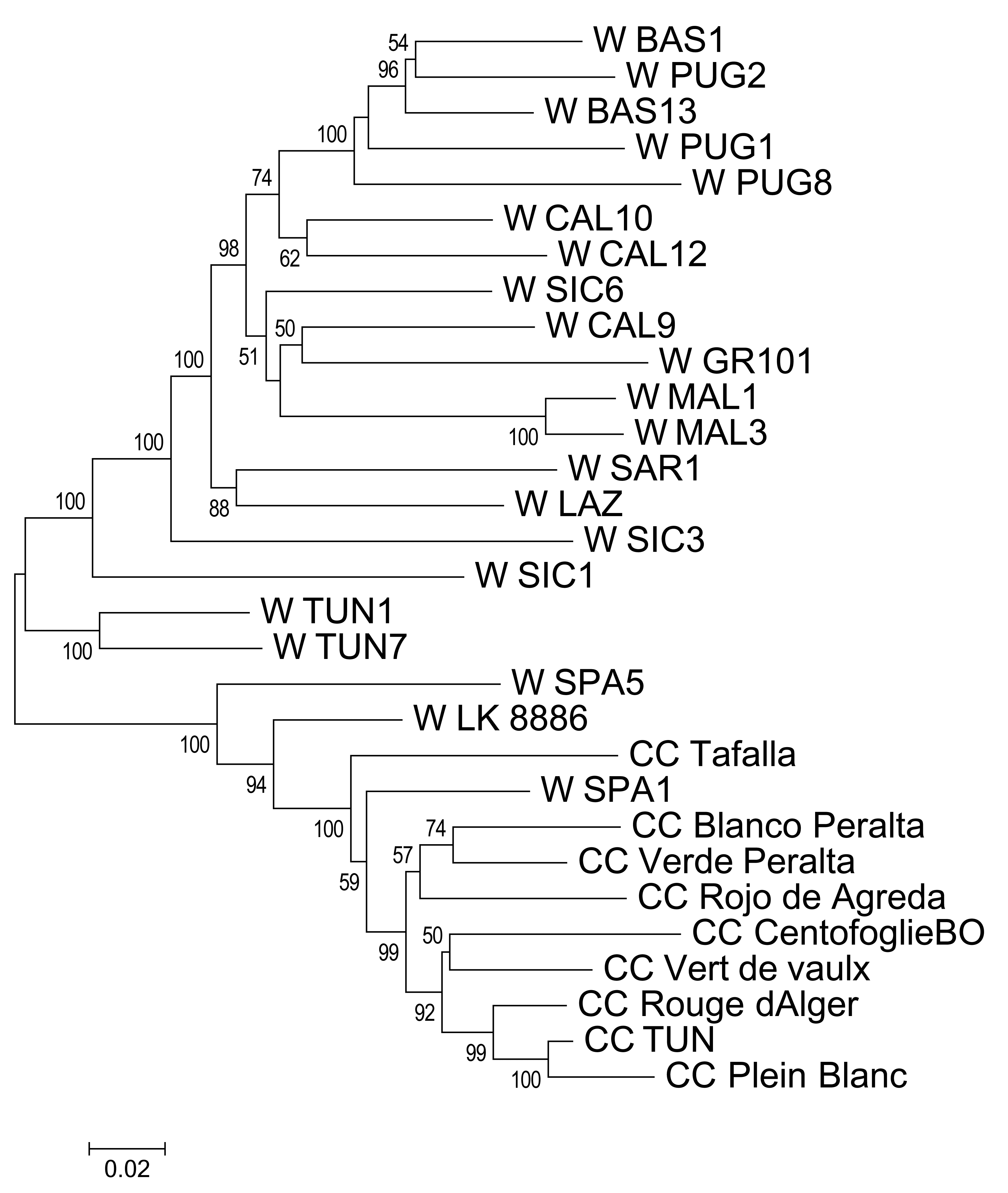

Supplement: S6 Fig — Number on tree branches indicate bootstrap values (≥ 50). (TIF) [file pone.0205988.s008.tif]
